# Platinum Nanoparticle-embedded Porous Diamond Spherical Particles as an Active and Stable Heterogeneous Catalyst

**DOI:** 10.1038/s41598-017-08949-0

**Published:** 2017-08-17

**Authors:** Takeshi Kondo, Takuji Morimura, Tatsumi Tsujimoto, Tatsuo Aikawa, Makoto Yuasa

**Affiliations:** 1 Department of Pure and Applied Chemistry, Faculty of Science and Technology, Tokyo University of Science, 2641 Yamazaki, Noda, Chiba 278-8510 Japan; 2 Research Institute for Science and Technology, Tokyo University of Science, 2641 Yamazaki, Noda, Chiba 278-8510 Japan

## Abstract

Platinum nanoparticle-embedded porous diamond spherical particles (PtNP@PDSPs), as an active and stable catalyst, were fabricated by spray-drying of an aqueous slurry containing nanodiamond (ND) particles, platinum nanoparticles (PtNP), and polyethylene glycol (PEG) to form ND/PtNP/PEG composite spherical particles, followed by removal of PEG and a short-time diamond growth on the surface. The average diameter of the PtNP@PDSPs can be controlled in the range of 1–5 μm according to the spray-drying conditions. The Brunauer-Emmett-Teller (BET) surface area and average pore diameter of the PtNP@PDSPs were estimated to be ca. 170–300 m^2^ g^−1^ and ca. 4–13 nm, respectively. When ND with the size of 20–30 nm was used, the size of PtNP in the PtNP@PDSP was almost unchanged at 5–6 nm even after high temperature processes and reuse test for catalytic reaction, showing stable supporting. The catalytic activity of the PtNP@PDSPs for the dehydrogenation of cyclohexane was higher than that for a Pt/C catalyst, which is attributed to the stable PtNP support by the three-dimensional packing of ND and efficient mass transfer via the interconnected through-hole pores in the PDSPs.

## Introduction

Metal nanoparticle (NP)-based heterogeneous catalysts are usually supported on a stable support material with a high specific surface area, such as alumina, silica, zeolite, and carbon, which enables the high specific surface area of the metal to be maintained with high catalytic activity. Core-shell type^1–6^ and yolk-shell type^7, 8^ catalysts have recently been developed for catalysts that exhibit high stability, catalytic activity, and selectivity. In these types of catalysts, metal NPs are embedded in a mesoporous material as a protective matrix with channels, which can avoid aggregation or sintering of the core metal NPs, even at high temperatures, and enables transfer of reactants/products through the channels. For example, a PtNP-mesoporous silica core-shell (PtNP@m-SiO_2_) catalyst was reported to have excellent stability for alkene hydrogenation^9^ and carbon monoxide (CO) oxidation^10^. For Pt-based core-shell catalysts, various shell materials including mesoporous carbon^11^, oxides^12^, polymers^13^, and metal-organic frameworks (MOF)^14^ have been used. Pd-based core-shell catalysts^15–24^ can be prepared in a similar way and can be used as, for example, a Suzuki coupling reaction catalyst with high catalytic activity and stability^22, 23^. Au-based core-shell catalysts have been reported to exhibit excellent stability for the reduction of 4-nitrophenol^25–30^. The Ag@CeO_2_ catalyst also exhibited highly chemoselectivity for the reduction of nitro compounds in the presence of C=C bonds^31^. Thus, the concept of core-shell catalysts is a powerful tool to design catalysts because various types of metal NP core materials and porous shell materials can be combined to produce new core-shell catalysts for specific reactions with high catalytic activity, selectivity and stability.

In this study, the concept of core-shell catalyst has inspired the development of a novel heterogeneous catalyst composed of PtNPs embedded in porous diamond spherical particles (PDSPs), a micrometer-sized diamond-based mesoporous material. Diamond is expected to be a promising shell material for a core-shell catalyst because of its extreme chemical and dimensional stability. PDSPs can be fabricated from spherical aggregate of nanodiamond (ND) particles prepared by spray-drying of an ND particle slurry, and voids between packed ND particles can act as mesopores. In our previous report^32^, the size of the PDSPs could be controlled in the range of approximately 1–10 μm by adjustment of the atomization speed during the spray-drying process, and the pore size could be changed according to the primary ND particle size in the range of 4–10 nm. In addition, the surface of the PDSPs can be functionalized, such as by photochemical surface modification with terminal alkenes. The PtNPs embedded in a PDSP are expected to be tolerant to aggregation or sintering because they are immobilized by three-dimensional packing of the chemically stable ND particles. Access of reactant and product molecules to the active sites is enabled via the mesopores; therefore, PtNP@PDSP is expected to act as an active and stable heterogeneous catalyst in the same way as reported core-shell catalysts. In addition, the size of the PtNP@PDSPs is relatively large compared to the typical size of core-shell catalysts (<500 nm) and thus separation of the catalyst from the reaction solution should be easily accomplished. This paper presents the fabrication and characterization of the PtNP@PDSPs, and evaluation of the catalytic activity and stability for the dehydrogenation of cyclohexane as a model reaction.

## Results and Discussion

### Fabrication and characterization of PtNP@PDSPs

**Figure 1:**
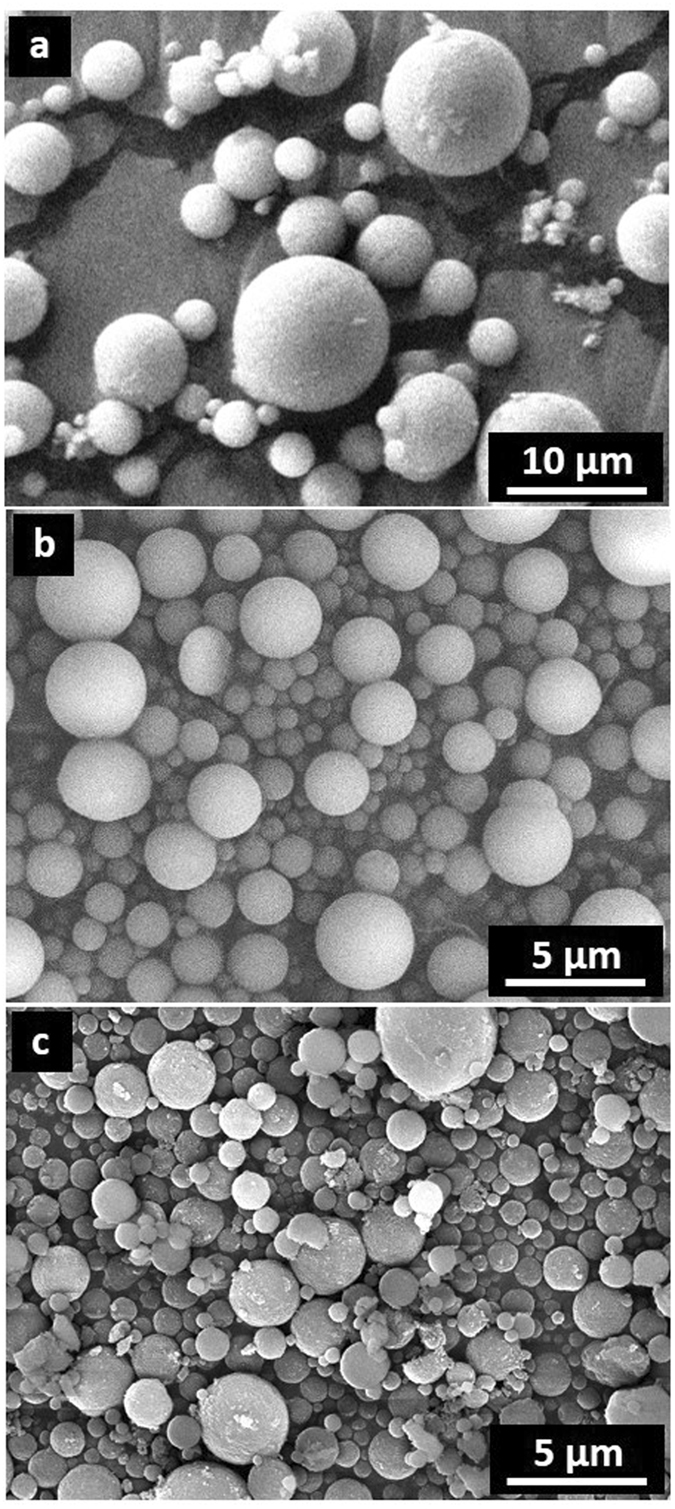
FE-SEM images of PtNP@PDSP-5 produced with air flow rates for atomization during spray-drying of (**a**) 246, (**b**) 473, and (**c**) 670 L h^−1^.

PtNP@PDSPs were fabricated by the same procedure as that previously reported^32^ for PDSPs, except for the presence of PtNP in the slurry. The PtNP@PDSPs produced were characterized comprehensively to confirm this assumption. Three different size of ND (ca. 5, 20 and 30 nm) was used for fabrication of PtNP@PDSP, and they are denoted here as PtNP@PDSP-5, -20, and -30, respectively. Figure 1 shows field emission scanning electron microscopy (FE-SEM) images of the PtNP@PDSP-5 fabricated under various conditions. Overall, the PtNP@PDSPs were confirmed to be micrometer-sized spherical particles. The average particle sizes of the PtNP@PDSPs with 0.1 wt% Pt were determined by dynamic light scattering (DLS) measurement to be ca. 1, 2, and 5 μm for spray-drying air flow rates of 670, 473, and 246 L h^−1^, respectively. The size of the particles prepared by spray-drying can be generally controlled according to the size of the droplets generated by atomization. Larger flow rates for atomization result in the generation of smaller droplets, and thus smaller PtNP@PDSPs are obtained. When the atomization flow rate was the same, the shape and size of the PtNP@PDSPs were almost identical, even if they contained different amounts of PtNP (0.1 and 3.0 wt% PtNP). Basically, similar result was obtained for PtNP@PDSP-20 and -30.

**Figure 2:**
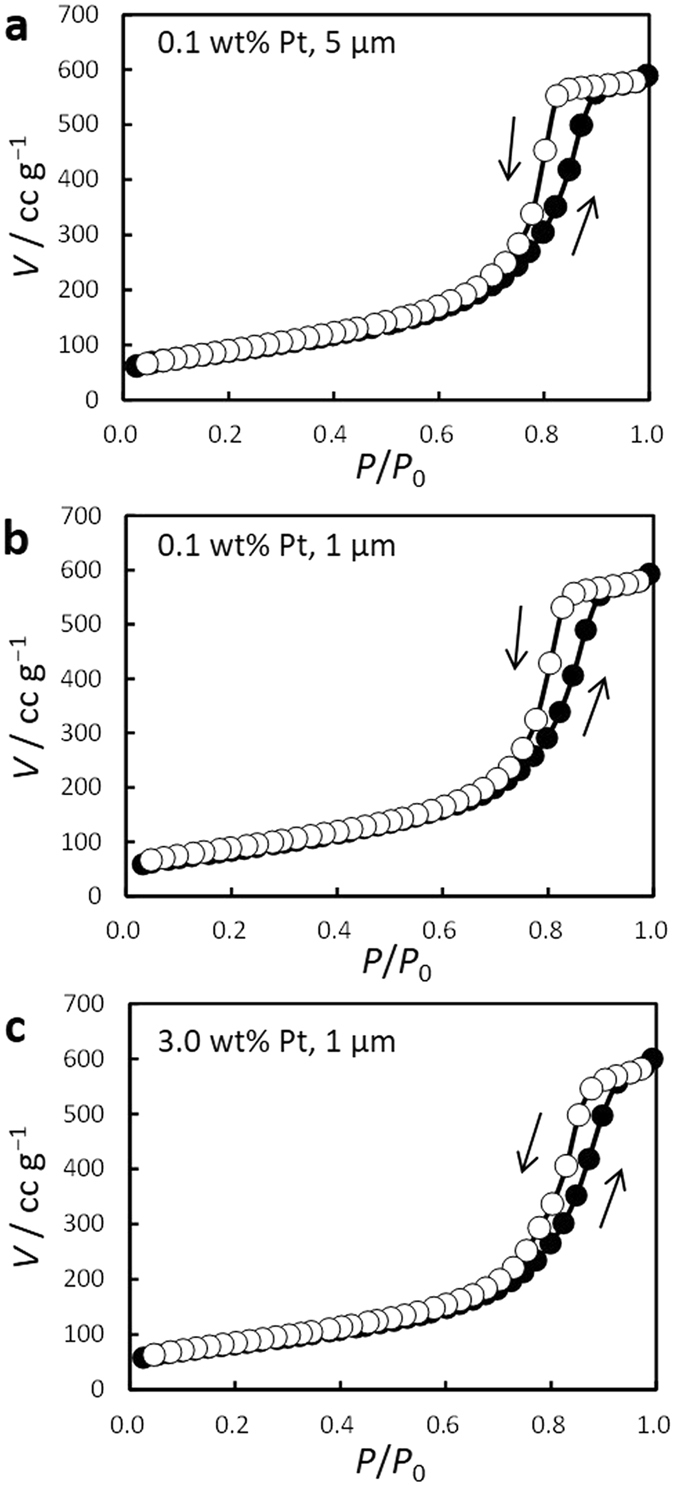
Nitrogen adsorption isotherms for PtNP@PDSP-5 with PtNP contents of (**a** and **b**) 0.1 and (**c**) 3.0 wt%. The average particle size was (**a**) 5 and (**b** and **c**) 1 μm.

Figure 2 shows nitrogen gas adsorption isotherms for PtNP@PDSP-5 produced with different atomization flow rates and PtNP contents. These isotherms had a hysteresis loop in the high-pressure region, which indicated that these materials were mesoporous. Table 1 lists the pore properties of the PtNP@PDSPs, including the Brunauer-Emmett-Teller (BET) surface area, total pore volume, and average pore diameter calculated from analysis of the isotherms by the Barrett-Joyner-Hallender (BJH) method. The pore properties were basically identical and independent of the atomization air flow rate or PtNP content. The pores in the PDSPs are based on the voids of packed ND particles; therefore, the size of the PtNP@PDSPs should not affect the pore properties. On the other hand, size of the ND used affected the pore properties because the specific surface area of ND reflects that of PtNP@PDSP and interparticle space between NDs acts as pores^32^. The size of the PtNPs was similar to that of the ND particles, and the amount of PtNPs was rather small. This is considered to be the reason why the amount of PtNPs in the PtNP@PDSPs had little effect on the pore properties. This was also confirmed by the almost identical pore properties as that previously estimated for PDSPs (without PtNP)^32^.

**Table 1:** Pore properties of PtNP@PDSPs fabricated under various conditions.

Sample	PtNP content^*a*^/wt%	Spray flow rate/L h^−1^	Average diameter/μm	BET surface area/m^2^ g^−1^	Total pore volume/cm^3^ g^−1^	Average pore diameter/nm
PtNP@PDSP-5	0.1	246	5	319	0.91	11.4
	0.1	670	1	312	0.92	11.7
	1.0	670	1	310	1.00	12.9
	3.0	670	1	294	0.99	13.4
PtNP@PDSP-20	3.0	670	1	260	0.29	4.5
PtNP@PDSP-30	3.0	670	1	168	0.32	7.7

^a^Pt content with respect to ND in the ND/PtNP/PEG slurry.

**Figure 3:**
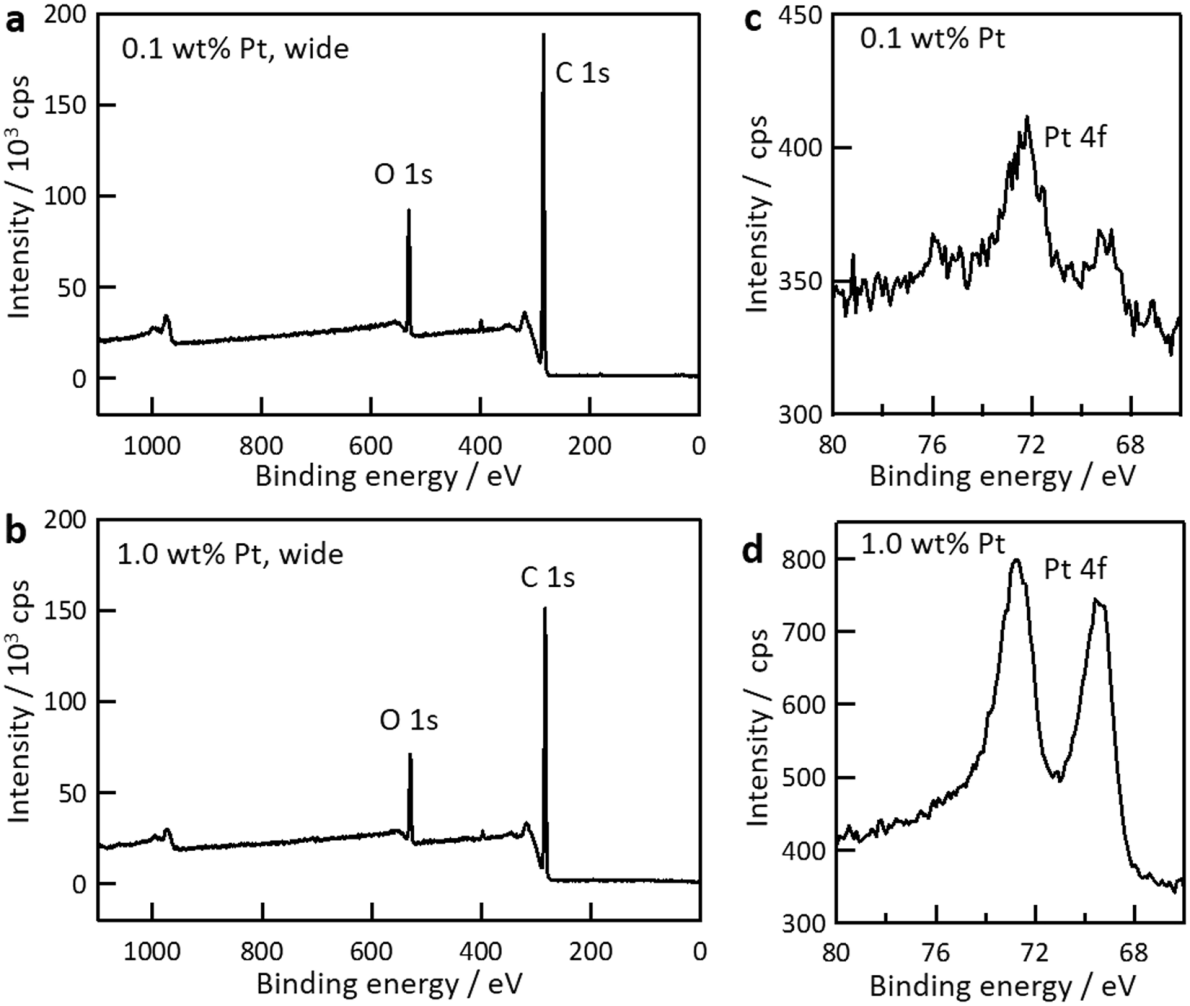
XPS spectra for PtNP@PDSP-5 with PtNP contents of (**a**) 0.1 and (**b**) 1.0 wt%. Pt 4 f spectra for PtNP@PDSP-5 with PtNP contents of (**c**) 0.1 and (**d**) 1.0 wt%.

The PtNP content in the PtNP@PDSP-5 was investigated using X-ray photoelectron spectroscopy (XPS). Figure 3 shows that the intensity of the Pt 4 f peak was greater for the PtNP@PDSPs with 1.0 wt% Pt than that with 0.1 wt% Pt. Analysis of the XPS data gave estimates of the Pt/(Pt + C) mass ratio of 0.1 and 1.3 wt% for the PtNP@PDSPs with 0.1 and 1.0 wt% Pt, respectively, which suggests that the PtNP content in the PtNP@PDSP could be controlled by the PtNP/ND ratio of the initial PtNP/ND/ polyethylene glycol (PEG) slurry. It should be noted that the elemental composition found from XPS measurements was that of the sample surface, considering the escape depth of photoelectrons (typically, several nanometers). For the fabrication of PtNP@PDSPs, PtNP/ND/PEG spherical particles were first prepared by spray-drying of the PtNP/ND/PEG slurry. The PtNPs and ND particles were considered to be dispersed homogeneously in the slurry and the drying process during spray-drying was rather rapid; therefore, the PtNPs and ND particles should also be dispersed homogeneously in the resultant PtNP/ND/PEG spherical particles. The subsequent processes, including thermal treatment and microwave plasma-assisted chemical vapor deposition (MPCVD), would not cause decomposition or effusion of the PtNPs. Thus, it is reasonable to consider that the distribution of PtNPs and ND particles in the PtNP@PDSPs was also homogeneous, and the PtNP/ND ratio would be identical to that of the initial slurry.

**Figure 4:**
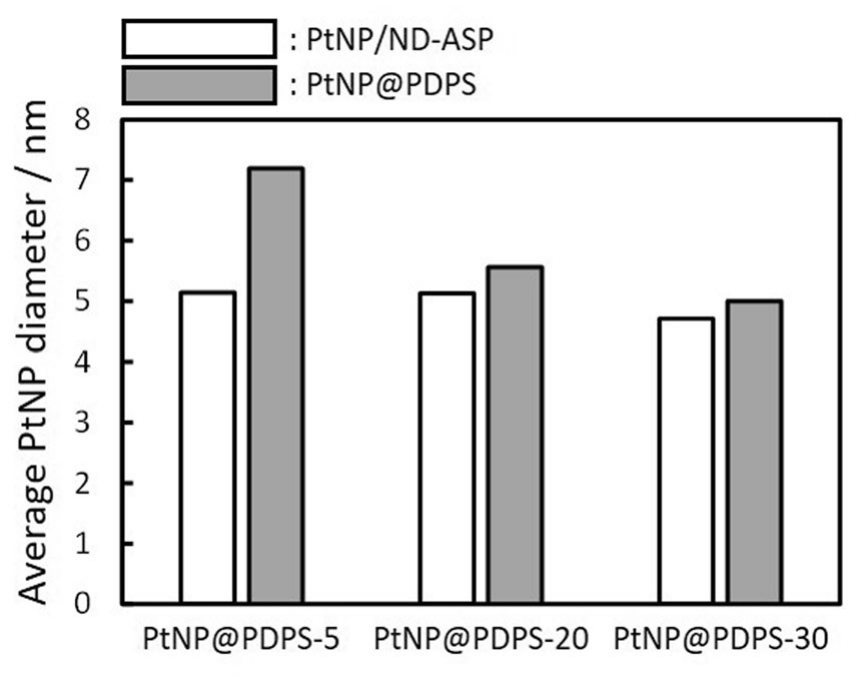
Average PtNP diameter in PtNP/ND-ASP and PtNP@PDSP. The PtNP diameter was estimated from Pt 220 peak of XRD pattern by Scherer equation.

Size of PtNP in the PtNP@PDSP was estimated from the the Pt 220 peak of X-ray diffraction (XRD) pattern by Scherrer equation (Fig. 4). In order to investigate the effect of high temperature processes on the PtNP size, average diameter of the PtNP in the PtNP/ND-ASP and PtNP@PDSP was compared. From the results, the PtNP size was found to increase after MPCVD and oxidation process when 5 nm ND was used for PDSP. Since the average pore dimeter of the PtNP@PDSP-5 was estimated by nitrogen sorption to be around 11–14 nm, which should represent interparticle space between aggregated ND with the size of ~50 nm^32^, the PtNP in the PDSP may migrate through the pore and cause sintering by the high temperature processes (MPCVD and oxidation). On the other hand, PtNP@PDSP-20 and -30 showed almost no change in the PtNP size even after the MPCVD/oxidation process. This result indicates that ND particle size should be a critical parameter for high durability to sintering of PtNPs. Thus, the metal NP support method by embedding in the PDSP is considered to be a useful method for the stable immobilization of metal NPs as primary particles. In this method, both the metal species and content can be controlled by design, and thus a variety of metal NP-supported heterogeneous catalysts could be created for a wide range of applications.

### Catalytic activity of PtNP@PDSP

The catalytic activity of the PtNP@PDSPs was estimated by comparison with a commercial Pt/C (Vulcan XC-72) catalyst using cyclohexane dehydrogenation (eq. 1) as a model reaction.

1 $${{\rm{C}}}_{{\rm{6}}}{{\rm{H}}}_{{\rm{12}}}({\rm{cyclohexane}})\to {{\rm{C}}}_{{\rm{6}}}{{\rm{H}}}_{{\rm{6}}}({\rm{benzene}})+{{\rm{3H}}}_{{\rm{2}}}$$

Cyclohexane is a liquid at atmospheric pressure and room temperature, and can be used as a hydrogen carrier because it can be reversibly dehydrogenated/hydrogenated using Pt-based catalysts^33^. Benzene, the main product of the cyclohexane dehydrogenation, can be determined easily in cyclohexane using gas chromatography (GC). Therefore, this reaction was used as a model reaction to estimate the catalytic activity of the PtNP@PDSP catalyst. The amount of active sites in the PtNP@PDSP catalyst was first evaluated with a catalyst analyzer based on CO pulse adsorption (Table 2). CO adsorbed to 1 g of PtNP@PDSP-5 with 3.0 wt% PtNP in the initial slurry was determined to be 1.42 cm^3^. Assuming that one CO molecule is adsorbed to a single surface Pt atom, the total Pt surface area in 1 g of the PtNP@PDSP-5 catalyst can be calculated to be 3.04 m^2^. The amount of PtNPs in the PtNP@PDSP-5 was evaluated by inductively coupled plasma atomic emission spectrometry (ICP-AES) to be 15.6 wt%, then the diameter of the PtNP can be calculated to be 14.3 nm. The significant increase of Pt content should be due to decrease of sp^2^ carbon content contained originally in the 5 nm ND, by the oxidation process after MPCVD. On the other hand, the Pt content in the PtNP@PDSP-20 and -30 evaluated by ICP-AES was found to be 3.2 and 4.2 wt%, respectively, which was almost consistent with the original Pt content to ND in the PtNP/ND/PEG slurry (3.0 wt%). The average diameter of the PtNP in these catalysts were calculated to be 6.2 and 6.1 nm, respectively. These results were somewhat larger than the particle size estimated by XRD. Since the diameter of the PtNP estimated by the CO adsorption data is based on the active surface area of Pt and the calculated diameter could be overestimated, so that the CO adsorption data are considered to be reasonable. Similarly, the amount of CO adsorbed to 1 g of the Pt/C catalyst was evaluated to be 3.27 cm^3^, which corresponds to PtNP diameter of 4.1 nm, taking the Pt content into consideration.

**Table 2:** Amount of CO adsorption, number of active sites, metal surface area, and average PtNP size of PtNP@PDSP and Pt/C catalysts estimated by the CO pulse adsorption method.

Catalyst	CO adsorption/cm^3^ g^−1^	Number of active sites/mol g^−1^	Metal surface area/m^2^ g^−1^-Pt	Metal surface area/m^2^ g^−1^-catalyst	Pt content/wt%	Average PtNP size/nm
PtNP@PDSP-5	1.42	6.34 × 10^−5^	19.5	3.04	15.6^a^	14.3
PtNP@PDSP-20	0.889	3.97 × 10^−5^	45.1	1.91	4.2^a^	6.2
PtNP@PDSP-30	0.679	3.03 × 10^−5^	46.1	1.46	3.2^a^	6.1
Pt/C	3.27	1.46 × 10^−4^	70	6.91	10.0^b^	4.1

^a^Evaluated by ICP-AES. ^b^Nominal data.

Based on these results, 100 mg of PtNP@PDSP-5, 159 mg of PtNP@PDSP-20 and 209 mg PtNP@PDSP-30 or 44 mg of the Pt/C was added to 40 mL of cyclohexane for reaction with an equivalent amount of active sites. After refluxing at 150 °C for 2 h, the catalysts were collected (by vacuum filtration for PtNP@PDSP and by centrifugation for Pt/C) followed by GC measurement of the solution to determine the concentration of benzene produced. The benzene concentration after reaction over the PtNP/PDSP-5, -20 and -30 catalyst was estimated to be 1.9, 1.8 and 1.5 mM, respectively, while that over the Pt/C catalysts was 0.56 mM, which confirms the higher catalytic activity of the PtNP/PDSP catalysts. Turnover frequency (TOF) of the reaction per active site was calculated to be 6.0, 5.7, 4.7 and 1.8 h^−1^, for PtNP@PDSP-5, -20, -30 and Pt/C catalysts, respectively.

It should be noted that the amount of benzene produced was smaller than the thermodynamic limit (650 mM), calculated on the basis of conversion value (7%) of cyclohexane dehydrogenation reaction at 150 °C^34^. However, since benzene production was not detected after reflux of cyclohexane without any catalyst (control), the presence of Pt should be essential for the production of a detectable amount of benzene at the condition. In addition, the effect of selective adsorption of benzene to the carbon support of Pt/C (Vulcan XC-72) should not be the reason for the small yield. The amount of adsorbed benzene can be estimated to be 1.2 μmol at most by the calculation with the molecular area of benzene monolayer (37 Å^2^/molecule)^35^ and specific surface area of Vulcan XC-72 (250 m^2^ g^−1^), and this value is much less than the detected benzene amount (22.4 μmol).

The amount of CO adsorbed on the PtNP@PDSP-5 after the reaction was estimated using the catalyst analyzer to be 1.29 cm^3^ g^−1^, which was almost the same as that before the reaction (1.42 cm^3^ g^−1^). In contrast, the amount of CO adsorbed on the Pt/C catalyst after the reaction was significantly decreased (1.23 cm^3^ g^−1^) from that before the reaction (3.27 cm^3^ g^−1^) (Table 2). These results indicate that the PtNPs were supported stably in the PDSPs even after the catalysis reaction, while the PtNPs supported on carbon were removed from the support and/or agglomerated on the support during the reaction. The PtNPs in the Pt/C catalyst are basically adsorbed on the surface of the carbon black support. In contrast, the PDSP support is physically rigid and provides stable immobilization of the PtNPs without removal or agglomeration under the reaction conditions for cyclohexane dehydrogenation and maintains the catalytic activity. It should be noted that cyclohexane dehydrogenation at Pt is known to be a structure-sensitive reaction^36^. However, in the present case, the Pt particle size estimated by XRD was larger than the particle size range where the particle size effect on the catalytic activity is apparent (1–4 nm)^36^. Thus, per-site activity at least in the initial stage should be comparable between PtNP@PDSP and Pt/C catalysts. Less activity of the Pt/C is considered to be due to the decrease in number of active site during the reaction.

**Figure 5:**
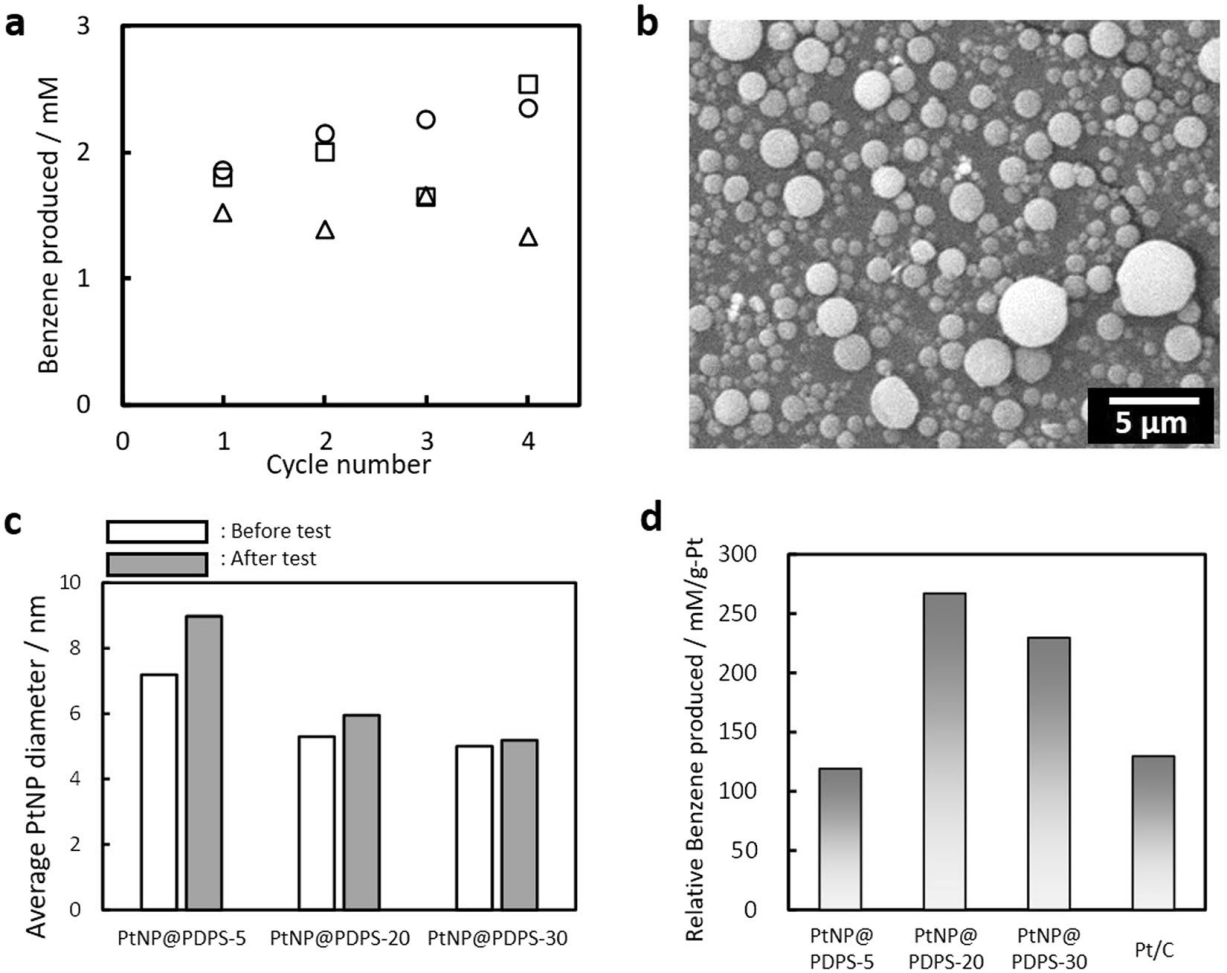
(**a**) Concentration of benzene produced by the dehydrogenation of cyclohexane over PtNP@PDSP catalysts as a function of cycle number for reuse tests. PtNP@PDSP-5 (○), -20 (□) and -30 (△). (**b**) FE-SEM image of the PtNP@PDSP catalyst after the reuse test. (**c**) Average diameter of PtNP in the PtNP@PDSPs before and after the reuse test. (**d**) Concentration of benzene produced per unit weight of Pt in the PtNP@PDSP and Pt/C catalysts.

The collected catalysts were reused for repeated reactions with fresh cyclohexane after heat treatment in air at 200 °C for 2.5 h for reactivation. Figure 5a shows benzene concentration after the reaction with reused catalysts up to four cycles. One possible cause for the variation of the data may be due to coke formation during the reaction. Actually, the catalytic activity was deteriorated after the reaction without the reactivation treatment. It should be also noted that the catalyst weight after the reuse test (four cycles) was 78–80% of the initial weight of the PtNP@PDSP catalysts by the collection loss. Although there are still limitations to discuss quantitative estimation, however, it can be conclude that the catalytic activity was substantially unchanged during the reuse test up to four cycles. The shape of the PtNP@PDSP catalyst from SEM observations was determined to be unchanged, even after the reuse tests (Fig. 5b). Increase of the average diameter of the PtNP estimated by XRD was found to be suppressed for PtNP@PDSP-20 and -30 after the reuse test, showing high stability of supporting (Fig. 5c). Figure 5d shows benzene concentration after the cyclohexane dehydrogenation reaction per unit weight of Pt in the catalyst. This result indicates that PtNP@PDSP-20 and -30 are more efficient than the Pt/C catalyst in terms of Pt usage. In addition, the PtNP@PDSPs are micrometer-sized particles that can be easily separated from the reaction solution by filtration. Furthermore, the catalyst can be packed in a column for application in a continuous reaction system. These properties of the PtNP@PDSPs are considered advantageous and superior to other NP-based heterogeneous catalysts, such as Pt/C and conventional core-shell type catalysts.

To achieve relatively high catalytic activity, despite the micrometer-sized particles, efficient mass transfer of the reactant/product through the mesopores in the PtNP@PDSPs is considered to be essential. The catalytic activity of PtNP@PDSP with different particle sizes was thus compared to confirm efficient mass transfer. In this investigation, 0.1 g of the PtNP@PDSP-5 catalyst (1.0 wt% PtNPs) with an average particle diameter of 1 or 5 μm was added to 5 mL of cyclohexane, followed by refluxing at 180 °C for 150 min. As a result, the benzene concentration after the reaction was almost the same for these PtNP@PDSPs with different particle sizes (Table 3). If the PtNPs in the PtNP@PDSP catalyst are assumed to be dispersed homogeneously in the particle and the PtNPs located near the particle surfaces contribute predominantly as active sites, then the catalytic activity should be higher for the smaller particle catalyst rather than the larger particle catalyst. The result showed almost no difference in the catalytic activity, which indicates that the PtNPs present in the center of the PtNP@PDSPs act as active sites. The average pore diameter of the PtNP@PDSP-5 was ca. 10 nm and the pores consisted of an interconnected through-hole network. Thus, the accessibility of reactants/products to/from the active sites in the PtNP@PDSPs though the pores would be relatively easy. When the catalytic activity was compared with different PtNP content PtNP@PDSP-5 (1.0 wt% and 3.0 wt%, average particle diameter: 5 μm), the benzene concentration after the reaction was proportional to the PtNP content, i.e., the amount of active sites. These results indicate that the PtNP content, which can be intentionally controlled according to the initial PtNP/ND ratio, as well as ND size, is the predominant factor for the catalytic activity, and the particle size does not affect the catalytic activity, which implies that even PtNPs located near the center of the particle can act effectively as active sites.

**Table 3:** Comparison of catalytic activity for PtNP@PDSP-5 catalysts with different PtNP contents and average particle sizes. The amount of PtNP@PDSP catalyst used for the test was 100 mg.

PtNP content/wt%	Average catalyst diameter/μm	Concentration of benzene produced/mM
1.0	5	45
3.0	5	115
1.0	1	44

## Conclusions

PtNP@PDSPs were fabricated by spray-drying of an aqueous slurry containing PtNPs and ND particles, followed by diamond growth with MPCVD. The PtNP@PDSPs are spherical particles and the average particle size can be controlled at 1–5 μm according to the flow rate for atomization during spray-drying. Nitrogen gas sorption analysis confirmed that the PtNP@PDSPs have mesopores with an average size of ca. 4–13 nm. The PtNP@PDSPs were fabricated from an agglomerate of PtNPs and ND particles formed by forced rapid drying of a droplet of the PtNP/ND slurry; therefore, the PtNPs could be embedded in the ND particles homogeneously without change of the PtNP/ND ratio. Especially, the PtNP@PDSP-20 and -30 showed high durability to sintering of the PtNP in the particle by the multiple thermal treatments including the MPCVD process at > 800 °C. The catalytic activity for cyclohexane dehydrogenation was higher for the PtNP@PDSP catalyst than for a commercial Pt/C catalyst. CO adsorption revealed that the total Pt surface area of the Pt/C catalyst decreased after the reaction, possibly due to removal and/or agglomeration of the PtNPs in the catalyst, which led to inactivation of the catalysis. In contrast, the PtNP@PDSP showed no significant change in the total Pt surface area after the reaction, which is considered to be due to the high stability of the rigid PDSP support. No significant difference in the catalytic activity was found for PtNP@PDSPs with different average particle sizes (1 and 5 μm) but with the same total amount of Pt, which suggests that even the PtNPs located at the center of the PtNP@PDSP act as effective active sites due to efficient mass transfer of the reactant/products via the interconnected through-hole network of the mesopores.

The method proposed in this study is expected to be a useful and versatile method for the production of metal NP-based heterogeneous catalysts. In principle, various types of NPs (metal, metal oxides, and their mixtures) can be embedded in PDSPs at arbitrary contents (at least up to 3 wt% in our study). Diamond is dimensionally stable in contact with any organic solvent and aqueous solution at any pH; therefore, metal NP-embedded PDSPs can offer an active and stable metal NP-based heterogeneous catalysts designed for a wide range of applications. In contrast to conventional core-shell type NP-based catalysts, the metal NP-embedded PDSPs are as large as micrometer-size, and can thus be easily separated from a reaction solution by low-energy techniques (filtration and sedimentation). In addition, such catalysts can be packed in a column for continuous reaction systems. Thus, metal NP-embedded PDSPs should be a useful concept for creation of active and stable heterogeneous catalysts.

## Methods

### Fabrication of PtNP@PDSPs

**Figure 6:**
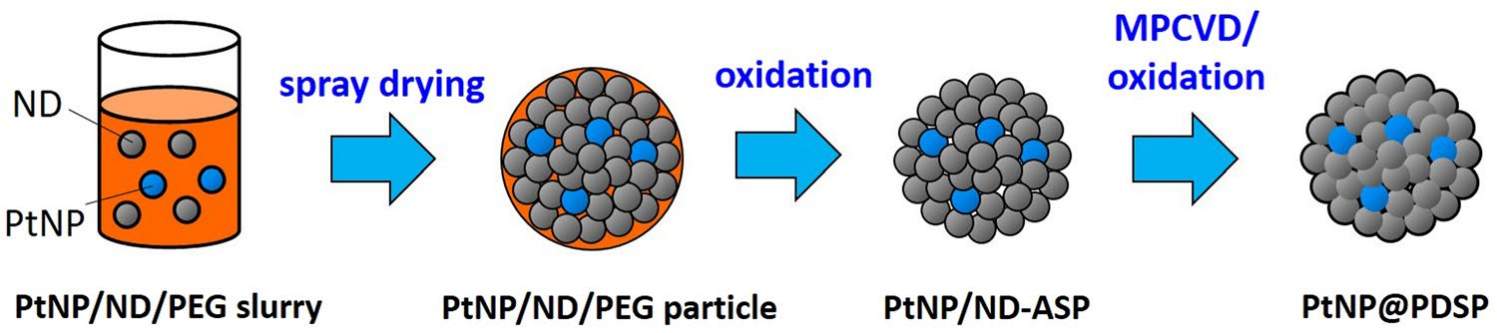
Schematic illustration for fabrication of PtNP@PDSP.

PtNP@PDSPs were fabricated using a procedure similar to that previously reported for the preparation of PDSPs^32^ (Fig. 6). An aqueous slurry of PtNPs, ND, and polyethylene glycol (PEG) was prepared by the addition of ND (Nanoamando, NanoCarbon Institute, nominal particle size 4.8 ± 0.7 nm; MD-20, Tomei Diamond, particle size 24 ± 6 nm; MD-30, Tomei Diamond, particle size 35 ± 9 nm) and a PtNP dispersion (Renaissance Energy Research, particle size 1–6 nm) to a 1.0 wt% aqueous solution of PEG, followed by ultrasonication. The ND concentration in the slurry was 5 wt%, and the Pt content was varied from 0.1 to 3.0 wt% of the ND content. The PtNP/ND/PEG slurry was then spray-dried (B-290, Büchi) to prepare PtNP/ND/PEG spherical particles. The air flow rates used for atomization during spray-drying was 246, 473, or 670 L h^−1^. Heat treatment of the spherical particles in air at 300 °C for 1 h in a muffle furnace allowed for the oxidative removal of PEG from the particles to obtain PtNP/ND-agglomerated spherical particles (PtNP/ND-ASPs). Short-time diamond growth on the PtNP/ND-ASP surface by microwave plasma-assisted chemical vapor deposition (MPCVD)^32^ was conducted to improve the mechanical strength of the particles. The as-deposited PtNP@PDSPs (AD-PtNP@PDSPs) were then subjected to oxidation in air at 425 °C for 5 h in a muffle furnace to remove sp^2^-carbon impurities^37^ and obtain PtNP@PDSPs.

### Characterization of PtNP@PDSPs

The shape and size of the PtNP@PDSPs were confirmed by observation with a field emission scanning electron microscope (FE-SEM; JSM-7600F, Jeol). The particle size distribution of the PtNP@PDSPs was estimated from dynamic light scattering (DLS; Nicomp 380, Particle Sizing Systems) measurements. Nitrogen gas adsorption isotherms, Brunauer-Emmett-Teller (BET) specific surface areas, total pore volumes, and average pore diameters were measured using a surface area and pore size analyzer (Autosorb-6, Yuasa Ionics). X-ray diffraction (XRD) patterns were recorded by an X-ray diffractometer (Ultima IV, Rigaku) with Cu Kα radiation, with an accelerating voltage of 40 kV and current of 40 mA. The Pt content in the PtNP@PDSPs was estimated using X-ray photoelectron spectroscopy (XPS; Axis-Nova, Kratos) or inductively coupled plasma atomic emission spectrometer (ICP-AES; ICPE-9000, Shimadzu). The metal surface area of the PtNP@PDSPs was evaluated on the basis of CO pulse adsorption onto the Pt surfaces using a catalyst analyzer (BELCAT-B, MicrotracBEL).

### Catalytic activity test of PtNP@PDSP

The catalytic activity of the PtNP@PDSPs was tested for the dehydrogenation of cyclohexane as a model reaction. Prior to use, the PtNP@PDSPs were pretreated in a quartz tube furnace at 350 °C for 3 h under a H_2_ gas atmosphere to activate the Pt surfaces. Cyclohexane dehydrogenation was conducted as follows, for example, 100 mg of the PtNP@PDSP catalyst was added to 40 mL of cyclohexane, followed by reflux at 150 °C for 2 h. After the reaction, the catalyst was collected by vacuum filtration. The filtrate solution was then sampled using gas chromatography (GC; GC-8A, Shimadzu) to determine the product benzene concentration. We confirmed that the benzene concentration was not changed by the filtration process.

## Electronic supplementary material

Supplementary Information
