# Supplementary material for: Platinum Nanoparticle-embedded Porous Diamond Spherical Particles as an Active and Stable Heterogeneous Catalyst
Source: Sci Rep. 2017 Aug 17;7:8651. doi: 10.1038/s41598-017-08949-0 (PMC5561195; doi:10.1038/s41598-017-08949-0)
Supplement: Supplementary file 1 — Supplementary Information [file 41598_2017_8949_MOESM1_ESM.pdf]

Supplementary Information for

Platinum Nanoparticle-embedded Porous Diamond Spherical Particles as an Active and Stable Heterogeneous Catalyst

Takeshi Kondo[1,2,*], Takuji Morimura[1], Tatsumi Tsujimoto[1], Tatsuo Aikawa[1], and Makoto Yuasa[1,2]

[1]Department of Pure and Applied Chemistry, Faculty of Science and Technology and [2]Research Institute for Science and Technology, Tokyo University of Science, 2641 Yamazaki, Noda, Chiba 278-8510, Japan

*t-kondo@rs.noda.tus.ac.jp

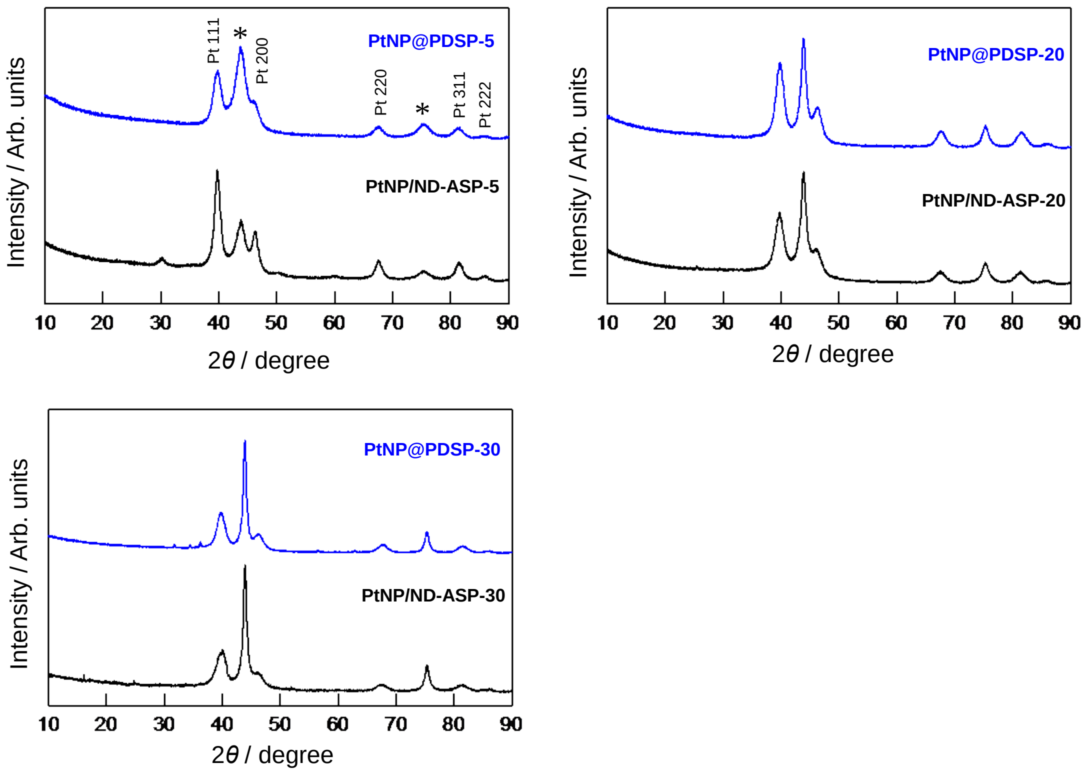

Figure S1. XRD patterns of PtNP@PDSPs and PtNP/ND-ASPs. Asterisk (*) indicates diamond.
